# Supplementary material for: A comprehensive investigation of the reaction behaviorial features of coke with different CRIs in the simulated cohesive zone of a blast furnace
Source: PLoS One. 2021 Jan 11;16(1):e0245124. doi: 10.1371/journal.pone.0245124 (PMC7799840; doi:10.1371/journal.pone.0245124)
Supplement: S1 File — (ZIP) [file pone.0245124.s001.zip › supporting files/Information of Coke A and Coke B.docx]

- **Information of Coke A and Coke B**

Both Coke A and Coke B were produced by ShaSteel coking Plant. Iron and Steel Research Institute of Shasteel provided the cokes for study.

Shagsteel Coking Plant is located in Zhangjiagang City, Jiangsu Province, with an annual output of 5.3 million tons of coke. There are two 7.63m top-charged coke ovens and six 6m top-charged coke ovens. And all the cokes are supplied to ShaSteel’s blast furnaces for ironmaking.

(1) Coke A produced by 7.63 m coke oven of ShaSteel Coking Plant

Coal blending scheme of coke A

| Coal Type | Name | Properties | | | | | Proportion/％ |
| --- | --- | --- | --- | --- | --- | --- | --- |
|  |  | A_d_/% | Vdaf/% | S_t,d_/% | G/% | Y/mm |  |
| Coking coal | JX | 10.2 | 21.4 | 1.1 | 84 | 15 | 11 |
|  | FJ | 10.5 | 22 | 0.55 | 88 | 16 | 18 |
|  | XN | 10 | 26 | 0.6 | 86 | 16 | 3 |
|  | TL | 10 | 20.8 | 1.12 | 82 | 15 | 9 |
|  | GYL | 10.0 | 25.2 | 0.50 | 88 | 16 | 5.2 |
|  | JX2 | 10.3 | 20.3 | 1.70 | 82 | 14 | 5.6 |
|  | LL | 10.4 | 22.2 | 1.7 | 83 | 14 | 5.6 |
| Fat coal | WH | 10.2 | 31.0 | 1.8 | 92.0 | 23.0 | 10.2 |
|  | XT | 10.5 | 31.5 | 0.5 | 92.0 | 26.0 | 4 |
|  | LX | 9.8 | 29.8 | 0.6 | 94.0 | 24.0 | 2.4 |
|  | ML | 10.5 | 26 | 1.2 | 95 | 26 | 9 |
| 1/3 coking coal | KZ | 8.0 | 36.1 | 0.6 | 80.0 | 12.0 | 3 |
|  | KZ2 | 7.3 | 35.99 | 0.56 | 85 | 13.5 | 5 |
| Lean coal | K10 | 10 | 18.2 | 0.22 | 82 | 9 | 2 |
|  | BKN | 6.5 | 18 | 0.8 | 85 | 12 | 7 |

(2) Coke B produced by 6 m coke oven of ShaSteel Coking Plant

Coal blending scheme of coke B

| Coal type | Name | Properties | | | | | Proportion/％ |
| --- | --- | --- | --- | --- | --- | --- | --- |
|  |  | A_d_/% | Vdaf/% | S_t,d_/% | G/% | Y/mm |  |
| Coking coal | JX | 10.3 | 20.3 | 1.7 | 84 | 15 | 16.0 |
|  | LL | 10.5 | 22.1 | 1.7 | 83 | 14 | 14.0 |
|  | GYL | 9.8 | 25.5 | 0.5 | 85 | 16 | 8.0 |
|  | XN | 10.2 | 25.5 | 0.6 | 85 | 16 | 2.0 |
|  | SJJX | 10.2 | 22 | 1.2 | 84 | 16 | 2.0 |
| Fat coal | LX | 9.8 | 28.5 | 0.6 | 93 | 23 | 2.0 |
|  | WH | 10.3 | 31.05 | 1.8 | 94 | 23 | 13.0 |
| 1/3 Coking coal | KZ | 8.2 | 36.2 | 0.6 | 80 | 13 | 8.0 |
|  | ZJD | 10 | 31 | 1.4 | 85 | 15 | 4.0 |
|  | DT | 8.4 | 36.3 | 0.6 | 77 | 12 | 16.0 |
| Lean coal | BS | 10.04 | 20.79 | 0.4 | 61 | 11 | 8.0 |
|  | K10 | 11.2 | 20 | 0.2 | 72 | 8 | 7.0 |

(3) Properties of coke A and Coke B

**Table 1 Properties of coke A and B**

| Sample | M_40_/% | M_10_/% | CRI/% | CSR/% | V_ad_/% | S_t,d_/% | A_d_/% |
| --- | --- | --- | --- | --- | --- | --- | --- |
| Coke A | 89.5 | 5.7 | 20.5 | 69.5 | 2.0 | 0.7 | 12.0 |
| Coke B | 85.2 | 7.2 | 28.6 | 65.5 | 2.5 | 0.7 | 12.0 |
