# Supplementary material for: A comprehensive investigation of the reaction behaviorial features of coke with different CRIs in the simulated cohesive zone of a blast furnace
Source: PLoS One. 2021 Jan 11;16(1):e0245124. doi: 10.1371/journal.pone.0245124 (PMC7799840; doi:10.1371/journal.pone.0245124)
Supplement: S1 File — (ZIP) [file pone.0245124.s001.zip › supporting files/supporting.docx]

**Supporting information**

**S1 Fig. The internal morphology of the reaction products**

S1 Figs. (a) and (b) show that slag and iron enter the inner holes of coke through open pores. The same phenomenon is found in deadman coke extracted from the blast furnace. The slag and iron entering the coke begin to corrode coke [47]. In the present experiment, slag and iron initially enter the inner pore of coke via the cohesive zone of the blast furnace. The porosity of coke is one of the factors that affect the CRI of coke. High porosity increases the slag content in coke. Therefore, reducing the porosity of coke is one of the methods that delay the degradation rate of coke.

[47]Chang ZY, Zhang JL, Ning XJ. Phase and mineral behavior of coke in cohesive zone, Fuel, 2019,253(1), 32-39.
