# Supplementary figures and images for: A comprehensive investigation of the reaction behaviorial features of coke with different CRIs in the simulated cohesive zone of a blast furnace
Source: PLoS One. 2021 Jan 11;16(1):e0245124. doi: 10.1371/journal.pone.0245124 (PMC7799840; doi:10.1371/journal.pone.0245124)

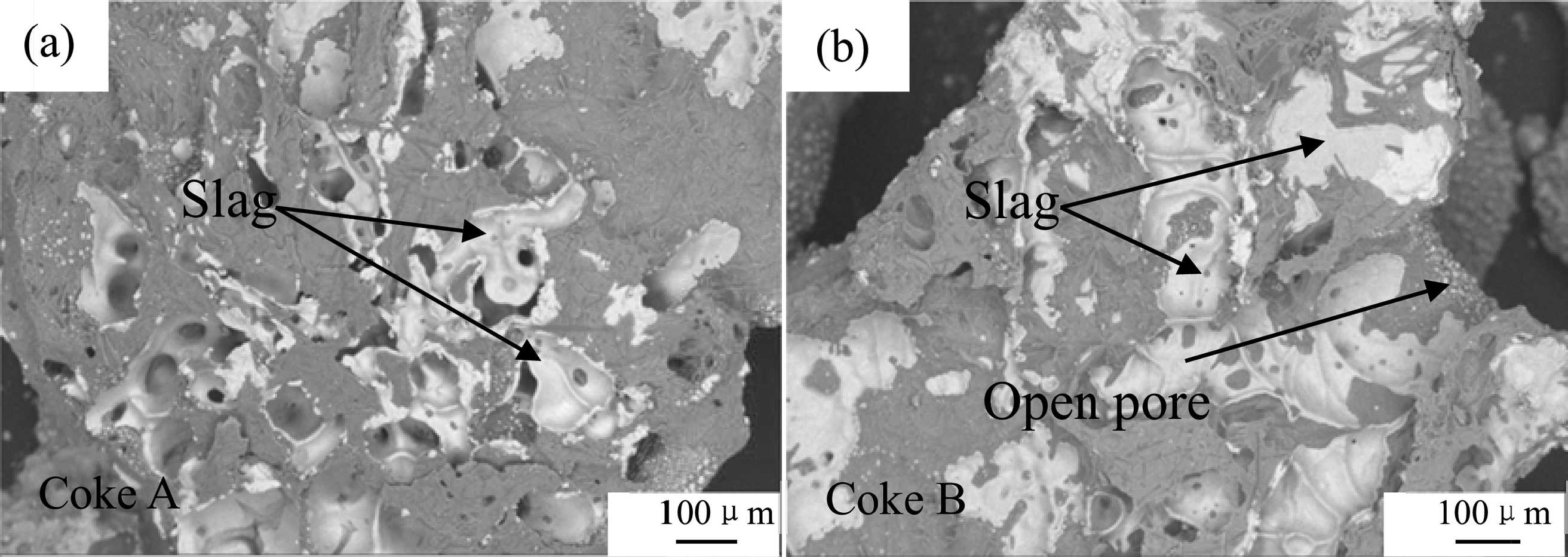

Supplement: S1 File — (ZIP) [file pone.0245124.s001.zip › supporting files/S1 Fig.tif]
