# Supplementary material for: A comprehensive investigation of the reaction behaviorial features of coke with different CRIs in the simulated cohesive zone of a blast furnace
Source: PLoS One. 2021 Jan 11;16(1):e0245124. doi: 10.1371/journal.pone.0245124 (PMC7799840; doi:10.1371/journal.pone.0245124)
Supplement: Attachment — Submitted filename: Reviewer suggestion.docx [file pone.0245124.s002.docx]

The authors present a study on the microstructural changes, gasification characteristics and reaction mechanism of coke with different CRI in a thermogravimetric analyzer. The fundamental characteristics were studied and tested by using different techniques, such as TGA, DTG, DTA, XRD, optical microscope, SEM and EDS etc. After reading this paper some questions arise that need be clarified by the authors.

1) In Table 1, the words CRI and CSR were explained in the manuscript, but the meaning of the words M_40_, M_10_, V_ad_, S_t,d_ and A_d_ was not appeared. Are these words familiar to professionals?

2) In Table 2,

Table 2 Chemical composition of sinter (wt. %)

| Elements | Fe | FeO | SiO_2_ | Al_2_O_3_ | CaO | MgO | P | S |
| --- | --- | --- | --- | --- | --- | --- | --- | --- |
| Sinter | 57.62 | 9.62 | 4.55 | 1.85 | 10.28 | 1.71 | 0.059 | 0.018 |

What is the meaning of letter T before Fe?

3) In page5 paragraph 2, “In Fig. 2, (002) and (100) peaks increase when coke is heated to 1350 ℃.” Should be “In Fig. 2, (002) and (100) peaks increase after coke is heated to 1350 ℃.”.

4) In Fig. 4, the scale should be added, even in the macrography.

5) References 1-23 are repeated.

6) The format of references should be unified, such as the references 4, 13, 16, 17, 23, 28, 36 and 41 should be use the full name of the journal, because the full name is used in other references; there is one more 2007 in Ref. 7; the name of the last author in Ref. 26.
