# Supplementary material for: A comprehensive investigation of the reaction behaviorial features of coke with different CRIs in the simulated cohesive zone of a blast furnace
Source: PLoS One. 2021 Jan 11;16(1):e0245124. doi: 10.1371/journal.pone.0245124 (PMC7799840; doi:10.1371/journal.pone.0245124)
Supplement: Attachment — Submitted filename: Response to Reviewers.docx [file pone.0245124.s003.docx]

Response to Reviewers

Dear two reviewers,

It is a great honor to receive the comments on this manuscript. Thank you for your patience. All the comments are very professional. I learned a lot from it. I have revised the manuscript to meet the requirements and the format of PLOS ONE. And I have chosen AJE for language editing of my manuscript. I hope that the resubmitted manuscript can meet the requirements of PLOS ONE.

If the manuscript still needs to be improved, please give feedback in time, and I will make corresponding modifications.

Thank you very much. The following is my reply to the two reviewers.

Reviewer #1:The authors present a study on the microstructural changes, gasification characteristics and reaction mechanism of coke with different CRI in a thermogravimetric analyzer. The fundamental characteristics were studied and tested by using different techniques, such as TGA, DTG, DTA, XRD, optical microscope, SEM and EDS etc. After reading this manuscript some questions arise that need be clarified by the authors.

1) In Table 1, the words CRI and CSR were explained in the manuscript, but the meaning of the words M40, M10, Vad, St,d and Ad was not appeared. Are these words familiar to professionals?

Answer：

Thank you for your question.

M_40_ represents the crushing strength of coke, and M_10_ represents the abrasion strength of coke. V_ad_ represents volatile content, S_t,d_ represents sulfur content, and A_d_ represents ash content. These words are the basic terms used to describe the performance analysis of the coke.

2) In Table 2,
Table 2 Chemical composition of sinter (wt. %)
Elements Fe FeO SiO2 Al2O3 CaO MgO P S
Sinter 57.62 9.62 4.55 1.85 10.28 1.71 0.059 0.018
What is the meaning of letter T before Fe?

Answer：

“T” represents “total”. So “TFe” means the total content of iron in the sinter.

3) In page5 paragraph 2, “In Fig. 2, (002) and (100) peaks increase when coke is heated to 1350 ℃.” Should be “In Fig. 2, (002) and (100) peaks increase after coke is heated to 1350 ℃.”.

Answer：

Many thanks!

This sentence is modified according to your requirements.

“In Fig. 2, (002) and (100) peaks increase after coke is heated to 1350 ℃.”.

4) In Fig. 4, the scale should be added, even in the macrography.
Answer：

Many thanks!

I have added the schale in Fig.4.

5) References 1-23 are repeated.
Answer：

I’m sorry for my mistake. I have deleted the repeated references. The format of references was modified according to the requirements of PloS one.

6) The format of references should be unified, such as the references 4, 13, 16, 17, 23, 28, 36 and 41 should be use the full name of the journal, because the full name is used in other references; there is one more 2007 in Ref. 7; the name of the last author in Ref. 26.

Answer：

Thank you! The name of the last author in the reference is the same as the name written in the reference. Please see the screenshot below for the specific name. The format of references was modified according to the requirements of PloS one.

Reviewer #2: Overall the manuscript “A Comprehensive Investigation on Reaction Behavior Features of Coke with Different CRI in the Simulated Cohesive Zone of a Blast Furnace” is a well described study investigating the reaction behavior of coke in blast furnace. I have few questions and comments which needs to be answered.

1. Equation 1 and two looks very shrink. It can be modified

Answer：

Many thanks! I enlarged the equation 1 and equation 2 appropriately.

2. Why elemental analysis was carried out in this study and what were the significance of analyzing only those elements which are presented in the table 3.

Answer：

The significance is to show the initial products of the reaction between coke and sinter. However, there is no element analysis explanation for table 3. To solve this problem, I added an explanation to the tables as shown below paragraph. I revised the original manuscript.

“In addition, Al, Si, Ti and Zr are found in the products. These elements, which are typically existing in the form of their oxides in the coke ash and sinter, form a part of the slag from blast furnaces. The generation and reduction of slag in the cohesive zone is are significantly affected by the burden containing iron. The sinter with a high content of alkalis is generally in the form of Cao-SO2-Al2O3-FeO-MgO. The element analysis in Table 3 tells shows that there are element differences between the slag in the present work and the primary slag from the blast furnace, indicatingwhich indicates that the ash and the slag have not been fully integrated”

3. Conclusion should be summarized in single paragraph rather than in points and should not be very long. It must reflect the findings and future perspectives of the study.

Answer：

Many thanks! I made the following changes to the conclusion of the article.

In this study, coke A and B were selected to study the reaction behavior of coke in the cohesive zone of a blast furnace through thermogravimetric analysis. Their microstructural changes and reaction mechanism were also analyzed. Under the cohesive zone temperature condition in the blast furnace, coke A and B undergo further pyrolysis and polycondensation. Direct and indirect reduction reactions occur between coke and sinter in the cohesive zone. The graphitization, gasification, and consumption rates of coke B are higher than those of coke A, indicating that the degradation speed of coke B is faster than that of coke A. The difference of CRI lies in the difference of molecular bond energy. According to the graphitization, gasification, and consumption rate research, it was found that CRI of coke plays a vital role on the deterioration of coke in cohesive zone of blast furnace. An in-depth understanding about the reactions of coke with different CRI in the high-temperature zone of blast furnace is still needed further research.

4. Figure 2 is not very clear. One cannot differentiate among different peaks.

Answer:

I adjusted the clarity of the picture. It’s easier to differentiate among different peaks than before.

5. Some of the latest references should be added like 2015 onward.

Answer:

Many thanks! I revisited the latest references and added relevant references.

6. Author should add some figures to the supplementary data as with so many figures in the main text will make manuscript very crowded.

Answer:

Many thanks. The Fig.12 is uploaded as a supporting file. I delete two figs of fig.11. And the manuscript now looks more concise and clear.

**Supporting information**

**S1 Fig. The internal morphology of the reaction products**

S1 Figs. (a) and (b) show that slag and iron enter the inner holes of coke through open pores. The same phenomenon is found in deadman coke extracted from the blast furnace. The slag and iron entering the coke begin to corrode coke [47]. In the present experiment, slag and iron initially enter the inner pore of coke via the cohesive zone of the blast furnace. The porosity of coke is one of the factors that affect the CRI of coke. High porosity increases the slag content in coke. Therefore, reducing the porosity of coke is one of the methods that delay the degradation rate of coke.

[47]Chang ZY, Zhang JL, Ning XJ. Phase and mineral behavior of coke in cohesive zone, Fuel, 2019,253(1), 32-39.
